# Supplementary material for: Texture and Flexural Fatigue Resistance Governed by Surface-Dependent Deformation and Recrystallization in the Copper Foils
Source: Nanomaterials (Basel). 2025 Dec 20;16(1):11. doi: 10.3390/nano16010011 (PMC12787428; doi:10.3390/nano16010011)
Supplement: Supplementary file 1 [file nanomaterials-16-00011-s001.zip › nanomaterials-4013698-supplementary.pdf]

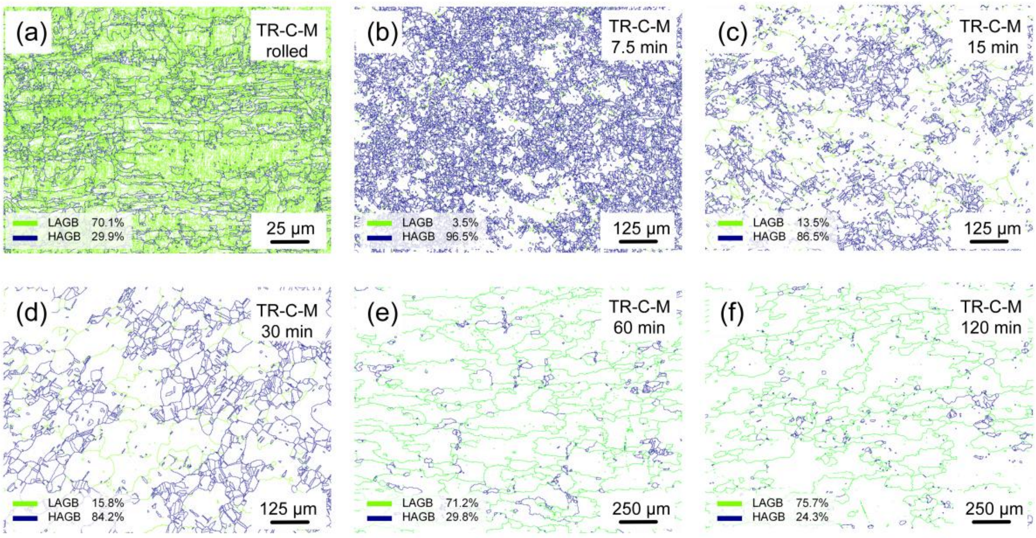

Figure S1: Distributions of Low- and High-Angle Grain Boundaries in the Central Layer (TR-C-M) after Different Annealing Times.

Table S1: Flexural fatigue life (N_f) of the TR-C-M layer in the cold-rolled condition and after annealing for different times.

Sample	1	2	3	4	5	Average
Rolled	9362	9694	9834	10094	11103	10017.4
7.5 min	72951	73084	81484	86584	104813	83783.2
15 min	42043	42778	45778	46078	51098	45555
30 min	32175	33990	35066	35980	36542	34750.6
60 min	27357	32416	35316	35816	35972	33375.4
120 min	23543	29540	31107	31420	31977	29517.4

Table S2: Flexural fatigue life (N_f) of the TR-O-B layer in the cold-rolled condition and after annealing for different times.

Sample	1	2	3	4	5	Average
Rolled	8534	8932	9108	9149	9677	9080
7.5 min	14553	14980	15771	16250	17801	15871
15 min	14751	15112	15647	16503	16933	15789.2
30 min	16235	16320	16554	17640	17734	16896.6
60 min	16873	17110	18481	19123	19873	18292
120 min	22295	22335	24200	24337	28343	24302

Table S3: Flexural fatigue life (N_f) of the TR-O-M layer in the cold-rolled condition and after annealing for different times.

Sample	1	2	3	4	5	Average
Rolled	12578	13789	14351	15123	15576	14283.4
7.5 min	15004	15690	16133	16840	17677	16268.8
15 min	17099	17110	17225	18021	20348	17960.6
30 min	17064	17102	19317	19480	21753	18943.2
60 min	19554	19607	19977	21820	21988	20589.2
120 min	22295	22335	24200	24337	28343	24302
